# Downregulation of the AU-Rich RNA-Binding Protein ZFP36 in Chronic HBV Patients: Implications for Anti-Inflammatory Therapy

**DOI:** 10.1371/journal.pone.0033356

**Published:** 2012-03-09

**Authors:** Wen-Jing Jin, Cai-Feng Chen, Hui-Yu Liao, Lu-Lu Gong, Xiao-Hui Yuan, Bin-Bin Zhao, Ding Zhang, Xia Feng, Jing-Jun Liu, Yu Wang, Guo-Feng Chen, Hui-Ping Yan, You-Wen He

**Affiliations:** 1 Key Laboratory of Systems Biology of Pathogens, Ministry of Health, Institute of Pathogen Biology, Chinese Academy of Medical Sciences & Peking Union Medical College, Dongcheng District, Beijing, China; 2 Center for Infection and Immunity, YouAn Hospital, The Beijing Capital Medical University, Beijing, China; 3 Fibrosis Noninvasive Diagnosis and Treatment Center, 302 Hospital, Beijing, China; 4 Department of Immunology, Duke University Medical Center, Durham, North Carolina, United States of America; Drexel University College of Medicine, United States of America

## Abstract

Inflammation caused by chronic hepatitis B virus (HBV) infection is associated with the development of cirrhosis and hepatocellular carcinoma; however, the mechanisms by which HBV infection induces inflammation and inflammatory cytokine production remain largely unknown. We analyzed the gene expression patterns of lymphocytes from chronic HBV-infected patients and found that the expression of ZFP36, an AU-rich element (ARE)-binding protein, was dramatically reduced in CD4^+^ and CD8^+^ T lymphocytes from chronic HBV patients. ZFP36 expression was also reduced in CD14^+^ monocytes and in total PBMCs from chronic HBV patients. To investigate the functional consequences of reduced ZFP36 expression, we knocked down ZFP36 in PBMCs from healthy donors using siRNA. siRNA-mediated silencing of ZFP36 resulted in dramatically increased expression of multiple inflammatory cytokines, most of which were also increased in the plasma of chronic HBV patients. Furthermore, we found that IL-8 and RANTES induced ZFP36 downregulation, and this effect was mediated through protein kinase C. Importantly, we found that HBsAg stimulated PBMCs to express IL-8 and RANTES, resulting in decreased ZFP36 expression. Our results suggest that an inflammatory feedback loop involving HBsAg, ZFP36, and inflammatory cytokines may play a critical role in the pathogenesis of chronic HBV and further indicate that ZFP36 may be an important target for anti-inflammatory therapy during chronic HBV infection.

## Introduction

Hepatitis B virus (HBV) is a non-cytopathic enveloped virus that consists of partially double-stranded DNA that encodes viral envelope proteins, core antigen (HBcAg), viral DNA polymerase, and the X protein. Approximately 350 million people are infected with HBV worldwide. HBV is highly prevalent in Southeast Asia, China, and Africa, where approximately 10% of the total population are HBV carriers [1]. During HBV infection, innate and adaptive immune cells are activated and produce various cytokines. These cytokines function in viral clearance but also induce both acute and chronic inflammation and cause liver tissue damage and cirrhosis [2]. Importantly, the defective immunity to HBV observed in chronic HBV patients may be due to dysregulated expression of pro- and anti-inflammatory cytokines [3], [4], [5], [6], [7]. However, the molecular mechanisms underlying the cytokine dysregulation in chronic HBV patients have not been determined.

Cytokine expression is subjected to regulation by adenosine/uridine (AU)-rich elements (ARE) and ARE-binding proteins. The AU-rich elements are key regulatory elements that control cytokine expression by inducing the degradation of many cytokine mRNAs [8], [9]. A number of mRNAs, nearly 15% of the human transcriptome, have an ARE in their 3′-untranslated region (UTR) [10], [11]. These mRNAs encode cytokines, chemokines, growth factors, and pro-inflammatory enzymes involved in inflammation and immune responses. AREs can be divided into several classes based on their sequences and modes of degradation [12]. ARE-binding proteins such as ZFP36, KSRP, and HUR recognize ARE mRNAs, affecting their stability and translation and controlling their mRNA decay [13].

ZFP36, also known as tristetraprolin (TTP), Nup475, TIS11, or G0S24, is a member of the zinc-finger family of proteins [14]. Many target cytokine and chemokine mRNAs for ZFP36 have been identified, including TNF-α, GM-CSF, IFN-γ, IL-2, IL-3, COX-2, VEGF, IL-6, IL-8, IL-10, IL-12, and CXCL1 [15], [16], [17], [18], [19], [20], [21], [22], [23], [24], [25], [26], [27], [28], [29], [30], [31]. ZFP36 is predominantly localized to the cytoplasm and binds to mRNAs containing class II AREs [32], [33]. Mice lacking the ZFP36 gene suffer from severe chronic inflammation, demonstrating the importance of ZFP36 as a negative regulator of inflammatory gene expression [34], [35]. The expression and function of ZFP36 are regulated by the p38 mitogen activated protein kinase (p38MAPK) signaling pathway [36]. p38MAPK can phosphorylate serine residues in ZFP36, impairing its ability to destabilize its target mRNAs.

In an analysis of differential gene expression, we found that ZFP36 mRNA expression was significantly reduced in lymphocytes, monocytes, and total PBMCs from chronic HBV patients. We also performed multiplex cytokine assays and found that multiple inflammatory cytokines were increased in the plasma of these HBV patients. The reduced expression of ZFP36 in total PBMCs and T lymphocytes from chronic HBV patients may contribute to the elevated levels of inflammatory cytokines in the plasma of these patients. To determine the effect of reduced ZFP36 expression, we performed siRNA-mediated silencing of ZFP36 in PBMCs from healthy donors. Silencing of ZFP36 resulted in increased expression of multiple inflammatory cytokines, most of which were also elevated in the plasma of chronic HBV patients. Importantly, we have identified IL-8, RANTES, and HBsAg as negative regulators of ZFP36 expression. These results suggest that a feedback loop involving HBsAg, IL-8, RANTES, and ZFP36 may both initiate and maintain the inflammatory environment in chronic HBV patients.

## Methods

### Patients

A total of 29 chronic HBV patients (22 men and 7 women) were recruited for this study. All patients were HBsAg- and anti-HBc-positive and HCV- and HIV-negative. Fifteen of the HBV patients (11 men and 4 women) were from YouAn Hospital (Beijing, China), and 14 of them (11 men and 3 women) were from 302 Hospital (Beijing, China). A total of 19 healthy donors (13 men and 6 women) were also recruited for this study. The characteristics of the patients and healthy donors are listed in Table 1. All human subjects provided informed written consent. All of the studies were approved by the Institutional Ethics Committees for Human Studies at YouAn Hospital and 302 Hospital.

**Table 1:** Characteristics of CHB patients and healthy donors.

Characteristic	Value	
	*HBV patients*	*Healthy donors*
Number	29	19
% Men	75.9	68.4
Age	35.9 (20–65)	39.5 (26–51)
HBV DNA	2.5×10^7^ (<500-2.0×10^8^)	-
ALT level (U/L)	180.9 (17.9–1223)	16.7 (8.6–45.7)
AST level (U/L)	125.1 (12.6–902)	41.7 (11.8–81.9)
TBIL level (µmol/L)	57.7 (4.7–425.1)	10.4 (6.1–21.2)

ALT, alanine aminotransferase; AST, aspartate aminotransferase; DBIL, direct bilirubin; TBIL, total bilirubin.

### PBMC isolation and transfection with siRNA

Fresh blood from HBV patients and healthy donors was diluted with phosphate-buffered saline (PBS, Hyclone, Logan, Utah, USA) containing 5% fetal calf serum (FBS, Invitrogen Gibco, Carlsbad, California, USA). PBMCs were isolated using Ficoll-Paque™ PLUS (GE Healthcare, Buckinghamshire, England) and transfected using an Amaxa® Human T Cell Nucleofector® Kit (Lonza, Cologne, Germany). The cells were cultured in 12-well plates containing 2 ml RPMI 1640 medium (Hyclone) supplemented with 10% FBS and 1–5 ng/ml human IL-7 (Biolegend, San Diego, California, USA) in a humidified 37°C incubator in the presence of 5% CO_2_ for 24 hours. Transfection was performed using a Nucleofector® Device (Lonza) with program V-024.

The siRNA sequences used were as follows: ZFP36, 5′-CGACGAUAUAAUUAUUAUA-3′ [36] and negative control, 5′-CCUACGCCACCAAUUUCGU-3′ (Bioneer, Daejeon, South Korea).

### Stimulation of PBMCs

Human PBMCs were cultured at 37°C in the presence of 5% CO_2_ in RPMI 1640 medium supplemented with 10% FBS. Ninety six-well plates (Costar Corning, Lowell, Massachusetts, USA) were coated with 200 µl/well of PBS containing 1 µg/ml anti-CD3 and anti-CD28 antibodies (eBioscience, San Diego, California, USA) by incubating the plates overnight at 4°C or for 2–3 hours at 37°C/5% CO_2_. The PBMCs were then seeded at a density of 1–1.5×10^6^ cells/ml and stimulated with anti-CD3/CD28 or soluble CpG ODN2006 at a final concentration of 10 µg/ml (5′-TCGTCGTTTTGTCGTTTTGTCGTT-3′, Invitrogen) for 1–3 days.

To assess T cell activation, the expression of CD25 and CD69 on CD4^+^ and CD8^+^ T lymphocytes was measured 24 hours after anti-CD3/CD28 stimulation. The PBMCs were suspended in a volume of 1 ml, labeled with 1 µl CFSE (carboxyfluorescein succinimidyl ester) 5 mM, (Sigma-Aldrich, St. Louis, Missouri, USA), and stimulated. The proliferation of the cells was assessed by examining the dilution of CFSE by flow cytometry. Apoptosis was assessed by Annexin V staining, and cell cycle status was determined by propidium iodide staining (Sigma). All results were collected using a BD FACS Canto™ II with corresponding antibodies (BD, Franklin Lakes, New Jersey, USA).

### Measurement of cytokine secretion

Plasma samples were collected from HBV patients and healthy donors by centrifuging fresh blood at 400 g for 5 minutes. For analysis of cytokine production by PBMCs, PBMCs were stimulated for 3 days beginning 24 hours after transfection, and supernatants were collected. All cytokines were measured by Luminex 200 (Millipore, Billerica, Massachusetts, USA) using a Bio-PlexTM Reagent Kit, Diluent Kit, and human Grp I Cytokine 27-Plex Panel (Bio-Rad, Hercules, California, USA) according to the manufacturer's instructions.

### In vitro treatment of PBMCs

Freshly isolated PBMCs from healthy donors were cultured with recombinant HBsAg purified from prokaryotic expression and kindly provided by the Academy of Military Medical Sciences, Beijing, China at a final concentration of 2 µg/ml. Cytokines (PeproTech, Rocky Hill, New Jersey, USA) were added to cell cultures at concentrations equivalent to the mean concentrations found in the plasma of HBV patients (IL-8: 40 pg/ml, RANTES: 30 ng/ml, and IFN-γ: 1 ng/ml). To assess the roles of various signaling pathways, PBMCs were plated at densities of 1–1.5×10^6^ cells/ml in 96-well plates and cultured for 20 hours in the presence of the following signaling inhibitors: SB203580, 40 µM (Sigma-Aldrich); H89-Dihydrochloride, 20 pM (Merck, Darmstadt, Germany); and Bisindolylmaleimide I, 20 nM (Merck). After treatment, PBMCs were collected and prepared for RNA extraction and real-time PCR analysis.

### RNA extraction and real-time quantitative PCR

Total RNA was isolated from PBMCs using an RNAqueous®-Micro Kit (Ambion, Austin, Texas, USA) and used in reverse transcription PCR (RT-PCR) reactions using Superscript® III First-strand Synthesis System for RT-PCR (Invitrogen, Carlsbad, California, USA). Real-time PCR was carried out with an ABI 7900HT instrument (Applied Biosystems, Foster City, California, USA) using SYBR Green Master Mix 2× (ABI) in a 20 µl reaction volume containing 0.2 µl of cDNA and 0.5 µl each of forward and reverse real-time PCR primers (10 mM). The PCR conditions were as follows: 1 cycle of 50°C for 2 min; 1 cycle of 95°C for 10 min; 40 cycles of 95°C for 15 s and 60°C for 1 min; and 1 cycle of 95°C for 15 s, 60°C for 20 s, and 95°C for 15 s to analyze the dissociation (melt) curves and determine the primer specificity. All data were normalized to the level of the 18s rRNA transcript. Primers were as follows: human 18s rRNA forward, 5′-TCAACTTTCGATGGTAGTCGCCGT-3′ and reverse, 3′-TCCTTGGATGTGGTAGC CGTTTCT-5′; ZFP36 forward, 5′-CATGGCCAACCGTTACACC-3′ and reverse, 3′-AGCGACAGGAGGCTCTCGTAC-5′; IL-8 forward, 5′-CTGGCCGTGGCTCTCTTG-3′ and reverse, 3′-CCTTGGCAAAACTGCACCTT-5′; RANTES forward, 5′-GACACCACACCCTGCTGCT-3′ and reverse, 3′-TACTCCTTGATGTGGGCACG-5′; IFN-γ forward, 5′-CCAACGCAAAGCAATACATGA-3′ and reverse, 3′-CGCTTCCCTGTTTTAGCTGC-5′ [37].

### Statistical analysis

All data are presented as the means ± SE. Data were analyzed using GraghPad Prism 5.00 software, and data with p-values<0.05 (t-test) were considered statistically significant.

## Results

### ZFP36 gene expression is downregulated in PBMCs of HBV patients

**Figure 1:**
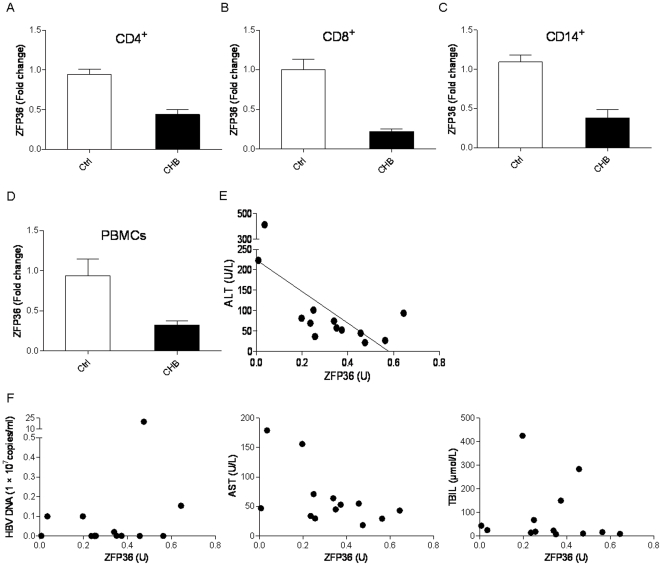
Downregulation of ZFP36 in T lymphocytes, monocytes, and total PBMCs of chronic HBV patients. ZFP36 mRNA expression was measured by qPCR in purified CD4^+^ T lymphocytes (**A**), CD8^+^ T lymphocytes (**B**), CD14^+^ monocytes (**C**), and total PBMCs (**D**) from chronic HBV patients (n = 13) and healthy donors (n = 9). ZFP36 expression in control cells was defined as 1.0. P values for all four groups were <0.01. **E.** Inverse correlation between ZFP36 expression in total PBMCs and ALT level (U/L) in chronic HBV patients (p = 0.011). **F.** Correlation between ZFP36 expression in total PBMCs and HBV DNA (p = 0.416), AST level (p = 0.056), and TBIL level (p = 0.770) in chronic HBV patients.

Although it is known that inflammation during chronic HBV infection is associated with liver pathogenesis and hepatocellular carcinoma development [38], the critical regulators of chronic inflammation during HBV infection have yet to be determined. We first performed an analysis of differential gene expression in CD8^+^ T cells from chronic HBV (CHB) patients and healthy donors (HD). As shown in Table S1, 527 genes displayed significantly different levels of expression in T cells from CHB than in those from HD (p<0.01). Our initial analysis found that ZFP36 mRNA expression in CD8^+^ T lymphocytes from CHB patients was reduced by 70% (p = 0.0006; n = 5) when compared to that in healthy donors. In contrast, the expression of two other members of the ZFP36 family, ZFP36L1 and ZFP36L2, was not changed (data not shown). We further confirmed this finding in CD4^+^ T lymphocytes (Figure 1A) (p = 0.0005), CD8^+^ T lymphocytes (Figure 1B) (p = 0.0045), CD14^+^ monocytes (Figure 1C) (p = 0.0071), and total PBMCs (Figure 1D) (p = 0.0029) from additional CHB patients (n = 13) and HD (n = 9) by qPCR. These results demonstrate that ZFP36 gene expression is markedly downregulated in multiple leukocyte populations and in total PBMCs from chronic HBV patients.

The clinical characteristics of all the patients and healthy donors enrolled in the study are listed in Table 1. The age and sex of the two populations were largely comparable. Interestingly, we found a significant negative correlation between ZFP36 expression in PBMCs and ALT levels in HBV patients (r = −0.675, R^2^ = 0.456, and p = 0.011) (Figure 1E). ZFP36 expression was weakly inversely correlated with AST levels (p = 0.056), and no correlations were found between ZFP36 expression and HBV DNA amount (p = 0.416) or TBIL level (p = 0.770) (Figure 1F). Given the known roles of ZFP36 in regulating cytokine production, these results suggest that the reduced expression of ZFP36 in the PBMCs of CHB patients may play a role in the chronic inflammation observed in these patients.

### Expression of cytokines in the plasma of CHB patients

**Figure 2:**
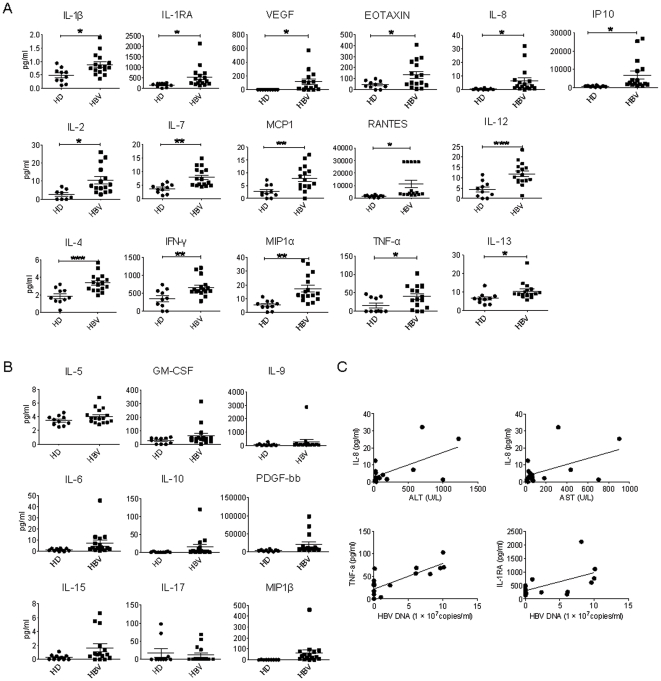
Plasma cytokine profiles of chronic HBV patients. **A and B.** Luminex results show the expression levels of 25 cytokines in the plasma of healthy donors (HD) (n = 10) and chronic HBV patients (n = 16). The expression levels (pg/ml) of cytokines that displayed significant differences between healthy donors and HBV patients are shown in A, and the expression levels of cytokines that did not display significant differences are shown in B. * p<0.05; ** p<0.01; *** p<0.001. **C.** Correlation between IL-8 and ALT levels (p<0.05); IL-8 and AST levels (p<0.05); TNF-α and HBV DNA levels (p<0.001); and IL-1RA and HBV DNA levels (p<0.05) in chronic HBV patients.

ZFP36 regulates cytokine expression by binding to AU-rich element sequences to destabilize cytokine mRNA. We thus hypothesized that the downregulation of ZFP36 in PBMCs of CHB patients contributed to an increased expression of inflammatory cytokines in chronic HBV patients. We first measured the plasma levels of 25 different cytokines. The expression of IL-1β, IL-1RA, IL-2, IL-8, IL-13, TNF-α, EOTAXIN, RANTES, VEGF, and IP10 in CHB patients (n = 16) was significantly higher than that in healthy donors (n = 10) (p<0.05) (Figure 2A). The differences in the expression levels of IL-7, IFN-γ, MCP1, and MIP1α (p<0.01) and of IL-4 and IL-12 (p<0.001) were even more pronounced (Figure 2A). Moreover, we observed a tendency toward increased levels of plasma GM-CSF, MIP1β, IL-5, IL-6, IL-9, IL-10, IL-15, IL-17, and PDGF-bb in CHB patients; however, these differences were not statistically significant (p>0.05) (Figure 2B). Despite the elevated levels observed for many cytokines in the plasma of CHB patients, microarray data showed that the mRNA levels of these cytokines were similar in T lymphocytes from CHB patients and healthy donors (data not shown). This finding suggests that the mRNA transcripts of these cytokines in T lymphocytes were not affected and that the elevated cytokine protein levels observed in CHB patients were due to post-transcriptional mechanisms.

Interestingly, we found a strong correlation between IL-8 and ALT levels (r = 0.611, R^2^ = 0.373, and p = 0.012) and between IL-8 and AST levels in CHB patients (r = 0.507, R^2^ = 0.258, and p = 0.045) (Figure 2C). Furthermore, we detected strong correlations between IL-1RA and HBV DNA levels (r = 0.559, R^2^ = 0.313, and p = 0.024) and between TNFα and HBV DNA levels (r = 0.788, R^2^ = 0.620, and p = 0.0003) (Figure 2C). These results suggest that inflammatory cytokines may directly influence liver pathology and that the levels of these cytokines may be correlated with HBV viral loads.

### Effect of silencing ZFP36 in PBMCs on cytokine production

**Figure 3:**
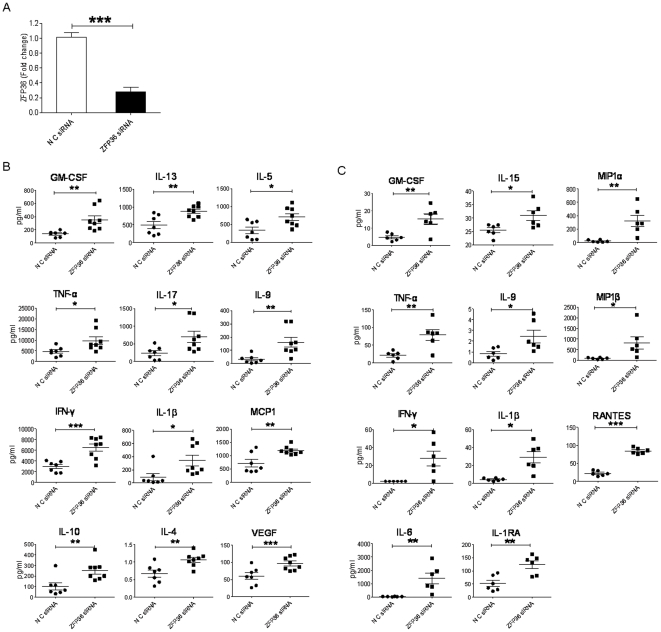
Effect of siRNA-mediated silencing of ZFP36 on cytokine production by T cells. **A.** Extent of ZFP36 silencing in PBMCs from healthy donors (n = 4). Total PBMCs from healthy donors were transfected by nucleofection with non-specific siRNA (NC siRNA) or ZFP36-specific siRNA (ZFP36 siRNA). qPCR analysis was used to quantify the expression of ZFP36 (p<0.001). **B.** Cytokine profiles in the supernatant of transfected and stimulated (anti-CD3/CD28) PBMCs. **C.** Cytokine profiles in the supernatant of transfected and stimulated (CpG ODN2006) PBMCs. The expression levels (pg/ml) of cytokines that displayed significant in control and ZFP36-silenced PBMCs are shown. * p<0.05; ** p<0.01; *** p<0.001. Data were obtained using cells from 4 different donors.

Although studies in the ZFP36-deficient mouse model have clearly demonstrated that ZFP36 negatively regulates inflammatory cytokine production [34], [39], the function of ZFP36 in primary human T lymphocytes is not clear. To directly assess the effect of reduced ZFP36 expression on cytokine production by primary human lymphocytes, we knocked down ZFP36 expression in PBMCs using RNAi. PBMCs from healthy donors were transfected by nucleofection with ZFP36-specific siRNA or control siRNA and then cultured for 48 hours. ZFP36-specific siRNA decreased ZFP36 mRNA expression by >75% in PBMCs from different donors (Figure 3A). Furthermore, we failed to detect ZFP36 protein expression in both wildtype and siRNA-treated PBMCs even when 5×10^6^ cells were used for Western blots, suggesting a low level of ZFP36 protein expression. The siRNA-treated human PBMCs were stimulated with anti-CD3/anti-CD28 or soluble CpG ODN2006 for 3 days, and cytokine production was analyzed. In contrast to the results of experiments performed on inbred mice, we observed large donor-to-donor variations in the production of cytokines by activated T lymphocytes and monocytes (Figure 3B, 3C). Silencing ZFP36 in PBMCs resulted in significantly enhanced production of TNF-α, IL-1β, IL-5, IL-17, GM-CSF, MCP1, IL-4, IL-10, IL-13, IL-9, IFN-γ, and VEGF following anti-CD3/CD28 stimulation (Figure 3B) and of TNF-α, IL-1β, IL-1RA, IL-5, GM-CSF, MIP1α, MIP1β, IL-15, IL-9, IFN-γ, and RANTES following CpG ODN2006 stimulation (Figure 3C).

**Figure 4:**
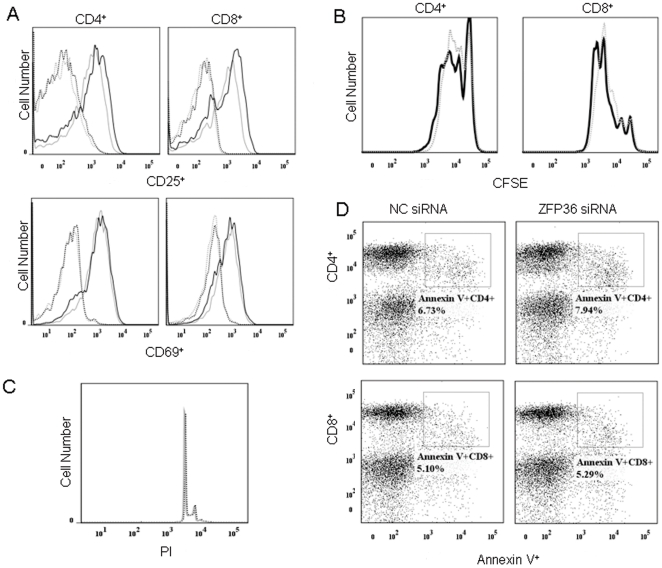
Effect of siRNA-mediated silencing of ZFP36 on T cell activation, proliferation, and survival. PBMCs from healthy donors were transfected by nucleofection with non-specific siRNA (NC siRNA) or ZFP36-specific siRNA (ZFP36 siRNA). **A.** Expression of CD25 and CD69 in CD4^+^ and CD8^+^ T cells was measured 24 h after stimulation with anti-CD3/CD28. Dotted lines: un-stimulated cells; solid lines: stimulated cells; grey lines: NC siRNA-transfected cells; black lines: ZFP36 siRNA-transfected cells. **B.** CFSE dilution of labeled CD4^+^ and CD8^+^ T lymphocytes. Dotted lines: cells transfected with NC siRNA; solid lines: cells transfected with ZFP36 siRNA. **C.** Cell cycle status of ZFP36 siRNA-transfected T cells. Cells were stained with PI. Solid lines: cells transfected with NC siRNA; dotted lines: cells transfected with ZFP36 siRNA. **D.** Apoptotic rates of ZFP36 siRNA-transfected T cells as determined by Annexin V staining. Percentages represent the frequency of Annexin V^+^ cells within the CD4^+^ and CD8^+^ T cell populations of PBMCs transfected with NC siRNA or ZFP36 siRNA.

We next addressed the possibility that the increased cytokine production in ZFP36 siRNA-treated T lymphocytes and monocytes was an indirect effect of the ability of ZFP36 to regulate cell activation, proliferation, or survival [13]. We examined whether siRNA-mediated silencing of ZFP36 in T lymphocytes affected T cell activation, proliferation, or survival. As shown in Figure 4, silencing ZFP36 in T lymphocytes had no apparent effect on their activation, proliferation, cell cycle progression, or apoptosis. These results suggest that ZFP36 specifically regulates cytokine production in primary human T lymphocytes.

The plasma cytokine profiles of CHB patients (Figure 2) and the supernatant cytokine profiles of ZFP36 siRNA-treated PBMCs stimulated with either anti-CD3/CD28 or CpG ODN2006 are compared in Table 2. Out of the 25 cytokines that were upregulated either in the plasma of CHB patients or in the supernatant of ZFP36 siRNA-treated PBMCs stimulated with anti-CD3/CD28 or CpG ODN2006, seven of them were shared between patient plasma and the supernatant of ZFP36 siRNA-treated PBMCs stimulated with anti-CD3/CD28: TNF-α, IFN-γ, MCP1, VEGF, IL-1β, IL-4, and IL-13. In addition, six cytokines were upregulated in both patient plasma and the supernatant of ZFP36 siRNA-treated PBMCs stimulated with CpG2006: TNF-α, IFN-β, MIP1α, RANTES, IL-1β, and IL-1RA. Among these elevated cytokines, TNF-α, IFN-γ, and IL-1β were upregulated in ZFP36 siRNA-treated PBMCs stimulated with either anti-CD3/CD28 or CpG ODN2006, suggesting that ZFP36 regulates the expression of these inflammatory cytokines in both T cells and monocytes. Furthermore, the ability of ZFP36 to modulate the expression of diverse cytokines in both T cells and monocytes suggests that reduced levels of ZFP36 may also result in enhanced cytokine production in other cell types.

**Table 2:** Comparison of cytokines increased in the plasma of CHB patients and the supernatant of PBMCs transfected with ZFP36 siRNA and stimulated with anti-CD3/CD28 or CpG.

	Plasma	Supernatant		ARE	Target of ZFP36
		*Anti-CD3/CD28*	*CpG 2006*		
TNF-α	*	*	**	Yes	Yes [15], [16]
IFN-ã	**	***	*	Yes	Yes [18], [19]
GM-CSF		**	**	Yes	Yes [17]
MCP1	**	**		Yes	Yes [19], [48]
MIP1α	**		**	Not determined	-
MIP1β			*	Not determined	-
EOTAXIN	*			Yes	Not determined
RANTES	*		***	Not determined	-
VEGF	*	***		Yes	Yes [24]
IP10	*			Yes	Not determined
PDGF-bb				Yes	Not determined
IL-1β	*	*	*	Yes	Yes [36]
IL-1RA	*		**	Not determined	-
IL-2	*			Yes	Yes [20]
IL-4	***	**		Yes	HuR Target [49], [50]
IL-5		*		Yes	Not determined
IL-6			**	Yes	Yes [25], [26]
IL-7	**			Yes	Not determined
IL-8	*			Yes	Yes [27]
IL-9		**	*	Not determined	-
IL-10		**		Yes	Yes [28], [29]
IL-12	***			Yes	Yes [30]
IL-13	*	**		Yes	HuR Target [51]
IL-15			*	Yes	Not determined
IL-17		*		Yes	ZFP36-like

* p<0.05;

** p<0.01;

*** p<0.001.

ARE data were obtained from the ARE-Containing mRNA Database from http://brp.kfshrc.edu.sa/ARED/. Bracketed numbers indicate references.

### HBsAg, IL-8, RANTES, and IFN-γ induce the downregulation of ZFP36 in PBMCs

**Figure 5:**
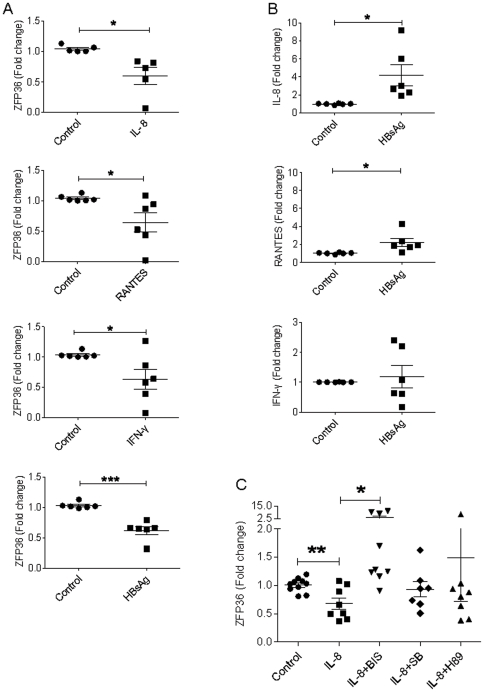
Reduced expression of ZFP36 in PBMCs after treatment with HBsAg, IL-8, RANTES, or IFN-γ. **A.** ZFP36 expression in PBMCs cultured with HBsAg (2 µg/ml), IL-8 (40 pg/ml), RANTES (30 ng/ml), or IFN-γ (1 ng/ml) for 20 hours. Shown are ZFP36 mRNA expression levels of samples from six healthy donors as measured by qPCR. **B.** Increased production of IL-8 and RANTES but not IFN-γ by HBsAg-stimulated PBMCs. PBMCs were cultured and tested as in A. **C.** Effect of bisindolylmaleimide I on IL-8-induced ZFP36 downregulation in PBMCs. PBMCs were cultured with IL-8 as in A in the presence of PKC inhibitor (BIS), PKA inhibitor (H89), or p38MAPK inhibitor (SB 203580). The expression of ZFP36 was measured by qPCR. *p<0.05; ** p<0.01; *** p<0.001.

The downregulation of ZFP36 expression in PBMCs of CHB patients may be mediated by HBV viral components and/or cytokines. To determine the factors that can induce ZFP36 downregulation, we tested the effects of various cytokines and HBsAg on ZFP36 expression in human PBMCs. PBMCs from healthy donors were cultured for 20 hours with 2 µg/ml HBsAg or with concentrations of cytokines equivalent to those found in the plasma of CHB patients. ZFP36 mRNA expression was significantly reduced in total PBMCs upon co-culture with HBsAg (p = 0.0001), IL-8 (p = 0.015), RANTES (p = 0.035), or IFN-γ (p = 0.037) (Figure 5A). This effect was specific, as 12 other cytokines that were elevated in CHB patients (TNF-α, IL-1β, IL-2, IL-17, IL-7, IL-12, IL-4, MIP1α, IP10, IL-13, EOTAXIN, and VEGF) did not affect ZFP36 expression (data not show).

To address the possibility that HBsAg may induce ZFP36 downregulation indirectly by inducing cytokine production, we analyzed IL-8, RANTES, and IFN-γ gene expression in HBsAg-stimulated PBMCs by qPCR. Indeed, HBsAg treatment of total PBMCs resulted in increased expression of IL-8 (p = 0.022) and RANTES (p = 0.025) but not of IFN-γ (p = 0.648) (Figure 5B). These data suggest that the presence of HBsAg during chronic HBV infection *in vivo* may enhance IL-8 and RANTES expression, resulting in the downregulation of ZFP36 expression and the upregulation of inflammatory cytokine production. Importantly, knockdown of ZFP36 in CpG stimulated monocytes resulted in enhanced RANTES production (Figure 3C), suggesting a feedback loop.

### IL-8-induced downregulation of ZFP36 in PBMCs can be blocked by bisindolylmaleimide I

IL-8 and RANTES are chemotactic for many cell types, including T lymphocytes, eosinophils, and basophils, and have many receptors. The most frequently studied receptors are the G protein-coupled serpentine receptors CXCR1 and CXCR2, which can induce signaling through a PKC-dependent pathway [40]. To better understand the mechanisms by which ZFP36 is downregulated during chronic HBV infection, we next studied the pathways that IL-8 uses to mediate its downregulation of ZFP36 expression.

PBMCs were cultured with IL-8 with or without pre-incubation with signaling pathway inhibitors including bisindolylmaleimide I (inhibitor of PKC signaling pathway), SB 203580 (inhibitor of P38MAPK), and H-89 dihydrochloride (inhibitor of PKA). ZFP36 expression was measured by qPCR. As expected, the expression of ZFP36 mRNA in PBMCs was reduced after incubation with IL-8 (p = 0.004). This IL-8-induced reduction in ZFP36 mRNA expression was inhibited by bisindolylmaleimide *I* treatment (Figure 5C). Importantly, two other inhibitors, H-89 dihydrochloride and SB 203580, did not significantly affect IL-8-induced downregulation of ZFP36. In addition, PBMCs incubated with bisindolylmaleimide I in the absence of IL-8 did displayed ZFP36 levels similar to those observed in untreated PBMCs, demonstrating that bisindolylmaleimide I alone does not affect ZFP36 expression (p = 0.217). Together, these results suggest that IL-8-mediated downregulation of ZFP36 in PBMCs likely occurs through the PKC signaling pathway.

## Discussion

A significant fraction of CHB patients develop liver cirrhosis and hepatocellular carcinoma, and the dysregulated expression of pro- and anti-inflammatory cytokines during the course of the disease may contribute to the pathogenesis of CHB [3], [4], [5], [6], [38], [41]. To develop an effective therapeutic strategy for CHB, it is essential to understand the molecular mechanisms underlying the dysregulated expression of pro- and anti-inflammatory cytokines in CHB patients. To achieve this goal, we have performed a differential gene expression analysis in lymphocytes from CHB patients and found that ZFP36 expression was decreased. Given the known roles of ZFP36 in regulating cytokine production in animal models and cell lines, we have further analyzed its function in primary human lymphocytes.

Our studies have resulted in several important findings. First, the expression of ZFP36, a key regulator of cytokine production, is reduced not only in CD4^+^ and CD8^+^ T lymphocytes, but also in CD14^+^ monocytes from CHB patients. Furthermore, total PBMCs also displayed significantly reduced ZFP36 mRNA expression, indicating an overall reduction of ZFP36 expression in hematopoietic cells. This is the first observation linking dysregulated ZFP36 expression with CHB infection. Second, ZFP36 expression is strongly inversely correlated with serum ALT levels and weakly inversely correlated with AST levels in CHB patients. These results are consistent with mouse genetic studies showing that ZFP36 negatively regulates the production of inflammatory cytokines including TNF-α, IL-1β, and IL-6 [34], [39]. Third, we have performed systemic cytokine expression profiling of plasma cytokine levels from CHB patients. Strikingly, our results indicate that out of the 25 analyzed cytokines, sixteen cytokines/chemokines/growth factors were significantly upregulated, and not a single cytokine was expressed at lower levels in the CHB patients than in healthy donors, indicating a general chronic activation of immune cells during CHB infection. Fourth, our RNAi experiments demonstrate that ZFP36 regulates a wide array of cytokines in PBMCs, and this effect is not mediated by changes in cell cycle progression or survival. Fifth, we have identified a feedback pathway involving HBsAg and IL-8 that mediates the downregulation of ZFP36 expression in PBMCs. Together, these results have provided important insight into the mechanisms controlling the dysregulated pro-inflammatory cytokine production in CHB patients.

Interestingly, we have observed not only a strong inverse correlation between the ALT levels and ZFP36 expression levels in CHB patients (Figure 1E), but also a strong positive correlation between the patients' ALT levels and plasma IL-8 levels (Figure 2C). Importantly, IL-8 is a potent inducer of ZFP36 downregulation (Figure 5A). These results are consistent with the notion that inflammatory cytokines such as IL-8 induce liver damage and promote further inflammation. In contrast, AST levels and ZFP36 expression levels were only weakly correlated, suggesting that AST and ALT levels may be regulated by different mechanisms in CHB patients.

Our results suggest that the reduced expression of ZFP36 in multiple cell types in CHB patients contributes to the elevated levels of inflammatory cytokines observed in these patients. Experiments in which ZFP36-silenced T lymphocytes or monocytes were stimulated with anti-CD3/CD28 or with CpG, respectively, clearly demonstrate that silencing of ZFP36 profoundly impacts cytokine production capacity in these cells. Of 25 cytokines tested, 12 were significantly elevated in T cells, and 11 were significantly elevated in monocytes. A comparison of the cytokines that were elevated in the plasma of CHB patients and those that were elevated in the supernatants of ZFP36 siRNA-treated PBMCs stimulated with anti-CD3/CD28 revealed that seven were shared: TNF-α, IFN-γ, MCP1, VEGF, IL-1β, IL-4, and IL-13 (Table 2). All seven of these cytokines have identified AREs and are established targets of ZFP36 or the AU-rich-binding protein HuR, suggesting a critical role for AU-rich RNA-binding proteins in regulating inflammation during CHB infection. Furthermore, a comparison of the cytokines that were elevated in the plasma of CHB patients and those that were elevated in the supernatants of ZFP36 siRNA-treated PBMCs stimulated with CpG revealed that six were shared: TNF-α, IFN-γ, MIP1α, RANTES, IL-1β, and IL-1RA. The levels of several other cytokines, including EOTAXIN, IP10, IL-2, IL-7, IL-8, and IL-12, were only elevated in the plasma of CHB patients but not in anti-CD3/CD28- or CpG-stimulated supernatants. This is likely due to the production of these cytokines by other types of cells, as the supernatants contained cytokines only from anti-CD3/CD28-simulated T lymphocytes or CpG-stimulated monocytes. Conversely, the levels of several cytokines were increased in the supernatants of ZFP36 siRNA-treated PBMCs stimulated with anti-CD3/CD28 (GM-CSF, IL-5, IL-9, IL-10, and IL-17) or with CpG (GM-CSF, MIP1β, IL-6, IL-9, and IL-15) but not in the plasma of CHB patients, suggesting that the production of these cytokines may be subjected to additional layers of regulation during CHB infection.

We next addressed the mechanism by which ZFP36 expression is reduced in the PBMCs of CHB patients by testing the effects of HBsAg and 15 cytokines that were elevated in CHB patient plasma on ZFP36 expression in total PBMCs from healthy donors. Our results indicate that HBsAg, IFNγ, IL-8, and RANTES significantly downregulated the expression of ZFP36. Furthermore, we found that HBsAg induced the expression of both IL-8 and RANTES in PBMCs. This observation is consistent with a very recent report showing that HBV induces IL-8 by directly activating its transcription [42]. The role of IL-8 in HBV pathogenesis has been well documented. The X protein of HBV activates IL-8 gene expression through NF-κB and C/EBP-like cis-elements [43], [44]. Furthermore, the significantly elevated IL-8 levels detected in CHB patients are associated with hepatic flares and liver damage [5], [45]. Our results suggest that one mechanism of IL-8-mediated liver damage is mediated through the downregulation of ZFP36 expression, which in turn causes the release of more inflammatory cytokines. This feedback loop likely helps to maintain the inflammatory environment during HBV infection. Importantly, our data indicate that RANTES is a target of ZFP36 suppression in monocytes and it is also a potent inducer of ZFP36 downregulation in PBMCs, suggesting a RANTES/ZFP36 feedback amplification of inflammation.

IFN-γ and TNF-α are hallmarks of the activation of HBV-specific CTLs, and both cytokines play major roles in the noncytolytic clearance of HBV infection in the liver [46]. ZFP36 downregulation in the PBMCs of CHB patients may lead to the upregulation of these two cytokines. It is puzzling that increased levels of TNF-α are associated with higher levels of HBV viremia. These results suggest that the enhanced levels of TNF-α and/or IFN-γ may play a detrimental role in the induction of liver damage. This is consistent with a report showing that elevated levels of inflammatory cytokines such as IL-8 and IFN-α are correlated with higher HBV viral loads and increased liver damage [5]. Although many of the cytokines that are elevated in CHB patients have been reported to display anti-viral activity, they failed to drive viral clearance in CHB patients. It is thus likely that viral clearance depends on a full restoration of exhausted T lymphocytes [47].

In summary, our studies suggest that ZFP36 is a key regulator of inflammation during CHB infection. HBV viral components such as HBsAg and the X protein upregulate IL-8 and other cytokines, including RANTES, to downregulate ZFP36 expression, which results in a de-repression of many inflammatory cytokines, including RANTES. To develop an effective therapy, this vicious inflammatory loop must be disrupted. Given its broad roles in controlling inflammatory cytokine production, ZFP36 may therefore prove to be an ideal therapeutic target. Future studies examining the link between dysregulated expression of ZFP36 and liver cirrhosis and hepatocellular carcinoma will thus provide critical information regarding the therapeutic value of targeting ZFP36 in chronic HBV infection.

## Supporting Information

Table S1 Human CD8^+^ T cells were purified using positive selection beads from three healthy donors and three chronic HBV patients. The purity was higher than 90% as assessed by flow cytometric analysis. Total RNA was extracted using mirVana kit. Total RNA (200 ng) was used for microarray hybridization. Differential gene expression was analyzed using one-way ANOVA. Genes that showed dramatically changed (fold change>2.0, p<0.05) were shown in the table.
(XLS)
